# Equine nutrition in the post‐operative colic: Survey of Diplomates of the American Colleges of Veterinary Internal Medicine and Veterinary Surgeons, and European Colleges of Equine Internal Medicine and Veterinary Surgeons

**DOI:** 10.1111/evj.13381

**Published:** 2021-01-09

**Authors:** April L. Lawson, Ceri E. Sherlock, Jo L. Ireland, Tim S. Mair

**Affiliations:** ^1^ Institute of Veterinary Science University of Liverpool Neston UK; ^2^ Bell Equine Veterinary Clinic Mereworth UK

**Keywords:** horse, colic, exploratory laparotomy, re‐feed, re‐water

## Abstract

**Background:**

Evidence is lacking concerning re‐introduction of feed and water following colic surgery.

**Objectives:**

To describe current approaches of European and American specialists to re‐introduction of feed and water in adult horses following surgical treatment of common intestinal lesions, assuming an uncomplicated recovery.

**Study design:**

Cross‐sectional survey.

**Methods:**

Electronic invitations, with a link to the online survey, were sent to 1,430 large animal specialists, including Diplomates of the ECVS, ACVS, ECEIM and ACVIM colleges.

**Results:**

The response rate was 12.6% including partial respondent data. Responses for each multiple‐choice question were between 123 and 178. Results are expressed as the percentage of the total number of responses and as a range where specific lesions are grouped together. Respondents reported that horses with large intestinal displacements were offered free choice water (63%‐65%) within 3 hours (55%‐63%), whereas horses with a small intestinal strangulating lesion were offered < 2 L water (64%‐74%) 12‐24 hours (28%‐34%) post‐operatively. Horses with a large colon displacement were offered feed within 3 hours of surgery (16%) with the majority offered feed 6‐12 hours (35%‐36%) post‐operatively. Horses with small intestinal strangulating lesions and small colon lesions were offered feed 24‐48 hours (34%‐42%) after surgery. Following small intestinal, small colon or caecal lesions, horses were re‐introduced feed in handfuls (79%‐93%) and initially with grass (41%‐54%). Horses with large colon displacements were mostly fed handfuls (49%‐50%) of forage initially, but a number of respondents would offer larger quantities such as a small bucket (35%‐37%) and predominantly of hay (50%‐51%).

**Main limitations:**

Low response rate. This study did not take into account common post‐operative complications that may alter the clinical approach.

**Conclusions:**

This post‐operative colic nutrition survey is the first to describe current clinical practice. Further research is required to investigate nutritional strategies in post‐operative colic cases.

## 1 INTRODUCTION

Feeding the post‐surgical colic case is predominantly led by clinicians’ experience and the lesion(s) identified. There is little published evidence regarding a ‘gold standard’ approach of what and when to feed, the amount and frequency. This highlights the importance of identifying the degree of variability in clinicians' current approaches to re‐introducing feed and water to the adult horse following colic surgery for common intestinal lesions. Although understandably, all cases are treated individually, it would be advantageous to gain information on the general approach for the uncomplicated case. While this would not establish a best practice approach, the information obtained from such descriptive research could provide the stimulus to generating future research with a greater emphasis on evidence‐based medicine.^1^, ^2^ Firstly, however, an understanding of current practice is needed.

It is probable that many horses in the post‐operative period following correction of gastrointestinal disorders would benefit from enteral nutrition (EN), and EN has the most encouraging impact in humans following gastrointestinal surgery. Human randomised control trials and meta‐analyses of early EN (<24 hours post‐operatively) demonstrate the potential beneficial effects on clinical outcomes including wound healing, anastomotic strength, gastrointestinal function and motility and length of hospital stay.^3^, ^4^, ^5^, ^6^, ^7^

Although we cannot directly extrapolate from humans to horses, it is believed that if the gastrointestinal tract is functional then EN should be encouraged. Positive indicators of a functional gastrointestinal system may include stable cardiovascular parameters, defaecation post‐operatively, reasonable appetite, good borborygmi, evidence of small intestinal motility or absence of distended, amotile loops of small intestines on ultrasound examination and absence of gastric reflux.^8^

The overall objective of this study was to gain an overview of the opinions and practices of European and American equine specialists. Within the human literature, this is an approach used when there is insufficient data for evidence‐based guidelines.^9^ This strategy has been adopted in other areas of equine medicine to identify and assess the opinions and practices of specialist clinicians in circumstances where definitive scientific evidence is lacking. This has provided a current international perspective on the views and present practices of equine veterinary specialists.^10^, ^11^

The specific aims of this study were 1) to report the different approaches favoured by European and American specialists to re‐feeding adult horses following surgical treatment of common intestinal lesions and 2) to identify the lesions that are more likely to undergo later re‐introduction of water and feed and return to full feed.

## 2 MATERIALS AND METHODS

The electronic questionnaire was created using web‐based proprietary software (SurveyMonkey Inc.). A preview of the survey was sent to a small group of four surgeons and internists (two of which were not involved in questionnaire design) to assess for practicality and for validation. The necessary amendments were made and subsequently an invitation to participate in the survey was delivered via email to all Diplomates of the European College of Equine Internal Medicine (ECEIM) and American College of Veterinary Internal Medicine (ACVIM) listed under Large Animal Medicine (total medicine specialists, n = 774), and the European College of Veterinary Surgeons (ECVS) and American College of Veterinary Surgeons (ACVS) listed under Large Animal Surgery (total surgery specialists, n = 656). Therefore, a total of 1,430 emails were sent to large animal listed specialists in medicine and surgery. The survey responses were obtained over an 8‐week period from August to October 2017. No reminders were sent to nonresponders.

The questionnaire (Data S1) was designed to enable completion within approximately 10–15 minutes, and consisted of 10 common surgical intestinal lesion scenarios. For each scenario, seven identical questions were asked, both closed‐ (eg multiple choice with tick boxes) and open‐ended (eg allowing comments) questions. This allowed for respondents to skip specific questions if they had not been exposed to a portion of the surgical scenarios contained in the questionnaire. The first two questions were aimed at identifying the timing and quantity of water that is first offered after surgery. The next three questions aimed to identify the timing, type and quantity of feed first offered. The sixth question was aimed at identifying over how many days, once re‐feeding had begun, clinicians returned horses to full feed. The final question for each scenario was to ascertain if any supplements would be used (eg electrolytes, prebiotics, probiotics, salt, mineral oil, etc.). At the end of the questionnaire, an open‐ended question was asked to ascertain clinicians’ approaches following cessation of post‐operative reflux (POR) to re‐introduction of water and feed.

### 2.1 Data analysis

Statistical analyses of the online survey results included descriptive analysis of respondent data. The mode was identified and the number of responders choosing that option was expressed as the percentage of the total number of responses and expressed as a range in the text when specific intestinal lesions were grouped together. Free‐text responses for open‐ended questions were categorised for analysis. Univariable logistic regression was performed when the categorical outcomes were re‐categorised into binary outcome variables for late re‐introduction of water (where late re‐introduction was defined as ≥12 hours post‐operatively) and feed (where late re‐introduction was defined as ≥24 hours post‐operatively), and for late return to full feed (defined as ≥4 days post‐operatively). Given that there is no clear normative category (intestinal scenario), the reference category for the logistic regression was determined based on the overall largest respondent rate for an intestinal scenario category and the same reference group was used for all the questions within the survey to maintain consistency. Significance was set at *P* ≤ .05. IBM SPSS 24 (IBM Corporation) was used for statistical analyses and prism 8 GraphPad (Prism, GraphPad Software) for generation of the graphs.

## 3 RESULTS

### 3.1 Respondent data

The response rate was 12.6% (180/1430). Respondents included members of ACVS (n = 75), ECVS (n = 38), ACVIM (n = 37), ECEIM (n = 12) and dual membership with ACVIM and ACVS (n = 1), ACVIM and ECEIM (n = 5) and ACVS and ECVS (n = 12). For some questions, there was a low level of item omission and some questions were only answerable conditionally on other responses; therefore, the denominators for the results vary between 123 and 178 for each multiple‐choice question and are reported throughout.

### 3.2 Re‐introduction of water

Respondents reported that horses with a large intestinal displacement would most commonly be offered free choice water (63%‐65%), with introduction of water within 3 hours following recovery from anaesthesia (55%‐63%). Respondents would re‐introduce water at <3 hours (34%‐35%) and as free choice (39%‐49%) for large colon torsion, caecal impaction with typhlotomy and small colon lesions. Respondents indicated that cases of caecal impaction with by‐pass had water re‐introduced at 3‐6 hours (30%) and with a volume of <2 L (44%). Horses with a small intestinal strangulating lesion were most commonly re‐introduced with <2 L water (64%‐74%) and 12‐24 hours (28%‐34%) after surgery (Table 1).

**Table 1:** A table displaying the raw data of the answers to the first two questions of the survey regarding re‐introduction of water: ‘When would you offer water following anaesthesia (assuming no reflux)?’ and ‘Volume of water offered first?’. Answers for all intestinal scenarios are displayed with the number of respondents and in italics are the percentage of respondents

Options	Intestinal scenarios – number of respondents (% respondents)																			
	Ileal impaction		Small intestinal strangulation ‐ no resection		Small intestinal strangulation resection J‐J		Small intestinal strangulation resection J‐C		Left dorsal displacement		Right dorsal displacement		≥360 degree large colon torsion		Caecal impaction ‐ typhlotomy only		Caecal impaction with by‐pass		Small colon strangulation and resection	
**When would you offer water following anaesthesia (assuming no reflux)?**																				
<3 hours	**81**	***46%***	40	*25%*	19	*12%*	18	*12%*	**91**	***63%***	**79**	***55%***	**49**	***35%***	**47**	***34%***	31	*24%*	**47**	***35%***
≥3 hours but < 6 hours	44	*25%*	33	*20%*	29	*19%*	29	*20%*	33	*23%*	43	*30%*	36	*26%*	41	*30%*	**38**	***30%***	44	*33%*
≥6 hours but < 12 hours	31	*17%*	35	*21%*	35	*23%*	32	*22%*	15	*10%*	18	*13%*	35	*25%*	31	*23%*	25	*20%*	22	*16%*
≥12 hours but < 24 hours	19	*11%*	**45**	***28%***	**52**	***34%***	**46**	***32%***	5	*3%*	4	*3%*	16	*12%*	10	*7%*	24	*19%*	18	*13%*
≥24 hours	3	*2%*	10	*6%*	18	*12%*	20	*14%*	0	*0%*	0	*0%*	3	*2%*	8	*6%*	9	*7%*	3	*2%*
TOTAL	178		163		153		145		144		144		139		137		127		134	
**Volume of water offered first?**																				
<2L	**71**	***40%***	**103**	***64%***	**113**	***74%***	**103**	***71%***	16	*11%*	21	*15%*	42	*30%*	43	*31%*	**56**	***44%***	37	*28%*
<10 L	41	*23%*	31	*19%*	18	*12%*	19	*13%*	35	*24%*	32	*22%*	43	*31%*	40	*29%*	34	*27%*	32	*24%*
Free choice	66	*37%*	27	*17%*	22	*14%*	23	*16%*	**93**	***65%***	**91**	***63%***	**54**	***39%***	**54**	***39%***	37	*29%*	**65**	***49%***
TOTAL	178		161		153		145		144		144		139		137		127		134	

Note

**Bold text** indicates the mode respondents answer. The results are displayed rounded to the closest whole number. J‐J, jejunojejunal anastomosis; J‐C, jejunocaecal anastomosis.

**Figure 1:**
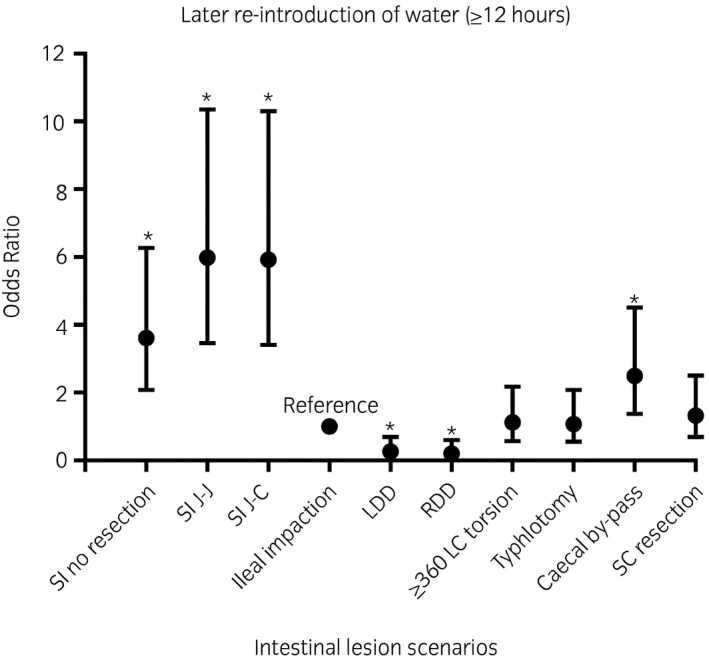
A graph plotting the odds ratio with the associated 95% confidence interval for later re‐introduction of water (≥12 hours) for each intestinal lesion scenario. * denotes significance (P ≤ 0.05). *SI, Small intestinal; J‐J, jejunojejunal anastomosis; J‐C, jejunocaecal anastomosis; RDD, right dorsal displacement; LDD, left dorsal displacement; SC, small colon*

Compared with the reference category of ileal impactions, the odds of late re‐introduction of water (≥12 hours) were greatest for small intestinal strangulating lesions that necessitated a resection, both jejunojejunal (odds ratio [OR] 5.98; confidence interval [CI] 3.46‐10.35; *P* < .001) and jejunocaecal (OR 5.92; CI 3.41‐10.30; *P* < .001) anastomoses, and small intestinal strangulating lesions without resection (OR 3.61; CI 2.08‐6.27; *P* < .001). Horses undergoing a by‐pass for caecal impaction had a greater OR for later re‐introduction of water (OR 2.49 [CI 1.37‐4.52; *P* = .003]) compared with typhlotomy only (OR 1.07 [CI 0.55‐2.09; *P* = .8]), with ileal impaction used as a reference category. Horses with a small colon lesion necessitating a resection and anastomosis (OR 1.32; CI 0.69‐2.51; *P* = .4) and large colon torsion (OR 1.12; CI 0.58‐2.17; *P* = .7) were comparable to the ileal impaction reference category. Compared with ileal impactions, large colon displacements had decreased odds (left dorsal displacement [LDD] OR 0.26 [CI 0.09‐0.69; *P* = .007]; right dorsal displacement [RDD] OR 0.20 [CI 0.07‐0.60; *P* = .004]) of late re‐introduction of water (Figure 1).

### 3.3 Re‐introduction of feed

Few respondents reported that they would offer horses with a large colon displacement feed <3 hours (16%) after surgery, with the mode response being 6‐12 hours (35%‐36%). Large colon torsion, caecal impaction and ileal impaction would be offered feed 12‐24 hours (27%‐34%) after surgery. Horses with small intestinal strangulating lesions and small colon lesions would be offered feed 24‐48 hours (34%‐42%) after surgery (Table 2).

**Table 2:** A table to display the raw data of the answers to the questions ‘When would you first offer feed following recovery from anaesthesia?’, ‘What type of feed would you offer when re‐introducing feed?’ and ‘Please estimate the quantity of feed you would offer initially’. Answers for all intestinal scenarios are displayed with the number of respondents and in italics are the percentage of respondents

Options	Intestinal scenarios – number of respondents (*% respondents*)																			
	Ileal impaction		Small intestinal strangulation ‐ no resection		Small intestinal strangulation resection J‐J		Small intestinal strangulation resection J‐C		Left dorsal displacement		Right dorsal displacement		≥360 degree large colon torsion		Caecal impaction ‐ typhlotomy only		Caecal impaction with by‐pass		Small colon strangulation and resection	
When would you first offer feed following recovery from anaesthesia?																				
<3 hours	10	*6%*	2	*1%*	1	*1%*	1	*1%*	23	*16%*	23	*16%*	12	*9%*	8	*6%*	5	*4%*	7	*5%*
≥ 3 hours but < 6 hours	31	*18%*	15	*9%*	8	*5%*	8	*6%*	40	*28%*	37	*26%*	23	*17%*	11	*8%*	7	*6%*	13	*10%*
≥ 6 hours but < 12 hours	47	*27%*	30	*19%*	18	*12%*	20	*14%*	**52**	***36%***	**51**	***35%***	34	*25%*	36	*26%*	22	*17%*	25	*19%*
≥ 12 hours but < 24 hours	**59**	***34%***	**56**	***35%***	50	*33%*	40	*28%*	26	*18%*	27	*19%*	**44**	***32%***	**37**	***27%***	**42**	***33%***	31	*23%*
≥ 24 hours but < 48 hours	27	*15%*	55	*34%*	**64**	***42%***	**60**	***41%***	3	*2%*	6	*4%*	23	*17%*	35	*26%*	39	*31%*	**45**	***34%***
≥ 48 hours	1	*1%*	4	*2%*	12	*8%*	16	*11%*	0	*0%*	0	*0%*	2	*1%*	10	*7%*	12	*9%*	13	*10%*
TOTAL	175		162		153		145		144		144		138		137		127		134	
What type of feed would you offer when re‐introducing feed?																				
Grass	**81**	***47%***	**82**	***51%***	**81**	***54%***	**76**	***53%***	47	*33%*	48	*34%*	**55**	***40%***	**62**	***46%***	**58**	***47%***	**54**	***41%***
Hay	33	*19%*	24	*15%*	17	*11%*	17	*12%*	**74**	***51%***	**72**	***50%***	49	*36%*	27	*20%*	21	*17%*	15	*11%*
Bran mash	15	*9%*	13	*8%*	14	*9%*	12	*8%*	9	*6%*	9	*6%*	7	*5%*	9	*7%*	9	*7%*	13	*10%*
Complete pelleted feed	34	*20%*	34	*21%*	30	*20%*	30	*21%*	10	*7%*	10	*7%*	19	*14%*	32	*24%*	29	*24%*	35	*27%*
Low residue diet	8	*5%*	7	*4%*	8	*5%*	8	*6%*	4	*3%*	4	*3%*	7	*5%*	5	*4%*	6	*5%*	15	*11%*
TOTAL	171		160		150		143		144		143		137		135		123		132	
Please estimate the quantity of feed you would offer initially.																				
Handfuls	**149**	***84%***	**142**	***89%***	**139**	***93%***	**131**	***93%***	**69**	***49%***	**70**	***50%***	**92**	***69%***	**108**	***81%***	**111**	***89%***	**104**	***79%***
Small bucket	20	*11%*	14	*9%*	8	*5%*	8	*6%*	52	*37%*	49	*35%*	35	*26%*	24	*18%*	13	*10%*	23	*18%*
Bucket	4	*2%*	2	*1%*	1	*1%*	1	*1%*	7	*5%*	8	*6%*	3	*2%*	1	*1%*	1	*1%*	4	*3%*
Haynet	4	*2%*	1	*1%*	1	*1%*	1	*1%*	12	*9%*	12	*9%*	4	*3%*	0	*0%*	0	*0%*	0	*0%*
TOTAL	177		159		149		141		140		139		134		133		125		131	

Note

**Bold text** indicates the mode respondents answer. The results are displayed rounded to the closest whole number. J‐J, jejunojejunal anastomosis; J‐C, jejunocaecal anastomosis.

Following various types of small intestinal, small colon and caecal lesions, respondents reported that they would re‐introduce feed in handfuls (79%‐93%) and initially with grass (41%‐54%). An alternative was a complete, pelleted diet (20%‐27%). Horses with large colon displacements were most often fed handfuls (49%‐50%) of forage initially, but compared with other lesions, a greater number of respondents would offer larger quantities such as a small bucket (35%‐37%) and predominantly of hay (50%‐51%). Similarly, respondents indicated that horses with a large colon torsion would be fed handfuls of feed (69%) and predominantly grass (40%), but a large proportion of respondents also fed hay (36%) after surgery (Table 2).

The most common respondent answer was that return to full feeds was delayed for horses with a small colon lesion, caecal impaction or small intestinal strangulating lesion necessitating jejunocaecal anastomosis (5 days) (26%‐32%). Horses with an ileal impaction, small intestinal strangulating lesion (without resection or jejunojejunal anastomosis), large intestinal displacement or torsion would be returned to full feeds sooner (3 days) (26%‐39%) (Table 3).

**Table 3:** A table to display the raw data of the answers to the question ‘Once re‐feeding has begun, over how many days do you aim for the horse to return to full feeds (assuming no reflux/complications)?’ of the survey regarding re‐introduction of feed. Answers for all intestinal scenarios are displayed with the number of respondents and in italics are the percentage of respondents

Options	Intestinal scenarios – number of respondents (*% respondents*)																			
	Ileal impaction		Small intestinal strangulation ‐ no resection		Small intestinal strangulation resection J‐J		Small intestinal strangulation resection J‐C		Left dorsal displacement		Right dorsal displacement		≥360 degree large colon torsion		Caecal impaction ‐ typhlotomy only		Caecal impaction with by‐pass		Small colon strangulation and resection	
Once re‐feeding has begun, over how many days do you aim for the horse to return to full feeds (assuming no reflux/complications)?																				
<1 day	1	*1%*	1	*1%*	0	*0%*	0	*0%*	5	*3%*	5	*3%*	0	*0%*	0	*0%*	0	*0%*	0	*0%*
1 day	11	*6%*	5	*3%*	3	*2%*	2	*1%*	22	*15%*	19	*13%*	7	*5%*	3	*2%*	0	*0%*	0	*0%*
2 days	32	*18%*	21	*13%*	11	*7%*	12	*8%*	38	*26%*	38	*26%*	21	*15%*	8	*6%*	8	*6%*	11	*8%*
3 days	**70**	***39%***	**52**	***32%***	**40**	***26%***	24	*17%*	**52**	***36%***	**55**	***38%***	**41**	***30%***	29	*21%*	26	*21%*	27	*20%*
4 days	28	*16%*	34	*21%*	33	*22%*	35	*24%*	16	*11%*	12	*8%*	26	*19%*	26	*19%*	23	*18%*	22	*17%*
5 days	24	*13%*	28	*17%*	36	*24%*	**37**	***26%***	9	*6%*	12	*8%*	31	*22%*	**43**	***32%***	**36**	***29%***	**41**	***31%***
6 days	10	*6%*	16	*10%*	23	*15%*	27	*19%*	2	*1%*	3	*2%*	10	*7%*	18	*13%*	22	*17%*	18	*14%*
>7 days	2	*1%*	4	*2%*	6	*4%*	7	*5%*	0	*0%*	0	*0%*	2	*1%*	9	*7%*	11	*9%*	14	*11%*
TOTAL	178		161		152		144		144		144		138		136		126		133	

Note

**Bold text** indicates the mode respondents answer. The results are displayed rounded to the closest whole number. J‐J, jejunojejunal anastomosis; J‐C, jejunocaecal anastomosis.

**Figure 2:**
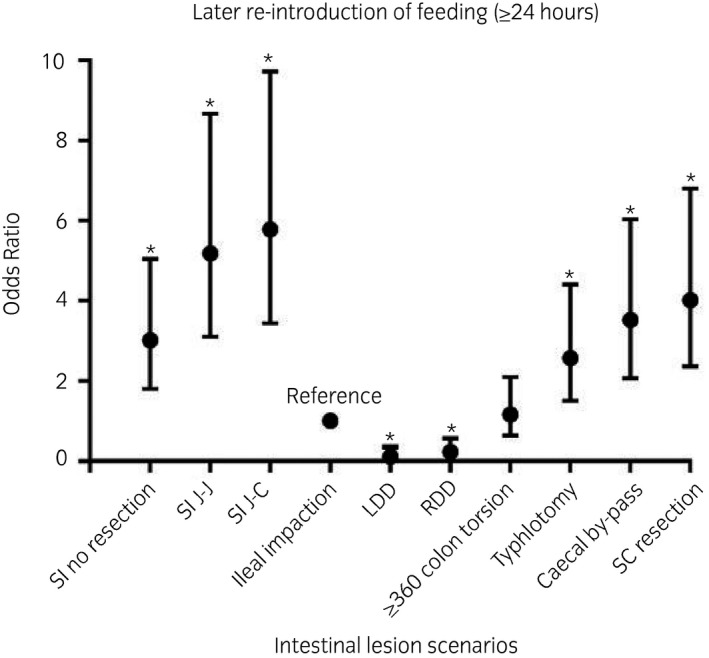
A graph plotting the odds ratio with the associated 95% confidence interval for later re‐introduction of feeding (≥24 hours) for each intestinal lesion scenario. * denotes significance (P ≤ 0.05). *SI, Small intestinal; J‐J, jejunojejunal anastomosis; J‐C, jejunocaecal anastomosis; RDD, right dorsal displacement; LDD, left dorsal displacement; SC, small colon*

Compared with horses with an ileal impaction, the odds of late re‐introduction to feed (≥24 hours) after surgery were greatest for horses with a small intestinal strangulating lesion that necessitated a resection and anastomosis (both jejunojejunal [OR 5.18; CI 3.10‐8.66; *P* < .001] and jejunocaecal [OR 5.78; CI 3.44‐9.72; *P* < .001]), and small colon lesion necessitating resection and anastomosis (OR 4.01; CI 2.36‐6.80; *P* < .001); followed by horses with a small intestinal strangulating lesion without resection (OR 3.01; CI 1.80‐5.04; *P* < .001), caecal impaction with by‐pass (OR 3.52; CI 2.06‐6.03; *P* < .001) and caecal impaction with typhlotomy only (OR 2.57; CI 1.50‐4.40; *P* = .001). Horses with a large colon torsion were re‐introduced feed at a comparable time to the reference category (OR 1.16; CI 0.64‐2.10; *P* = .6). Compared with horses with an ileal impaction, horses with a large colon displacement had decreased odds of late re‐introduction to feed (RDD OR 0.23 [CI 0.09‐0.57; *P* = .001]; LDD OR 0.11 [CI 0.03‐0.38; *P* < .001]) (Figure 2).

**Figure 3:**
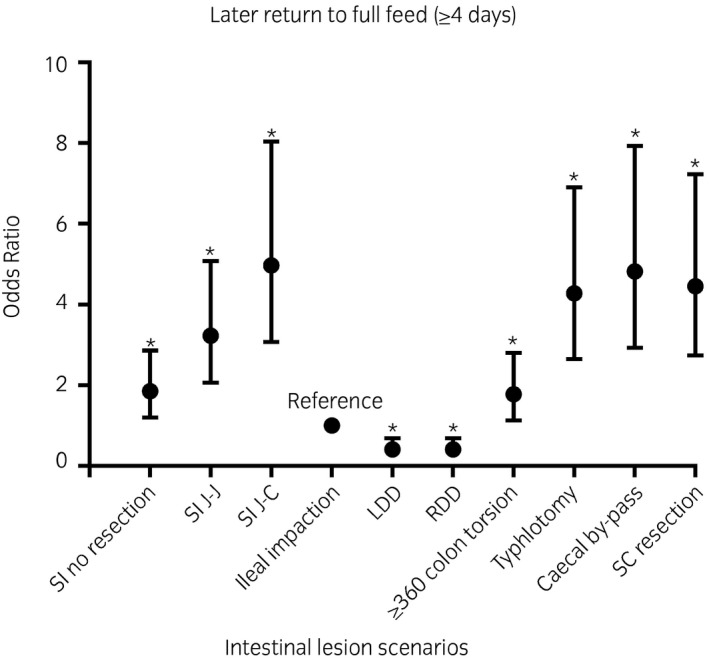
A graph plotting the odds ratio with the associated 95% confidence interval for later return to full feeds (≥4 days) for each intestinal lesion scenario. * denotes significance (P ≤ 0.05). *SI, Small intestinal; J‐J, jejunojejunal anastomosis; J‐C, jejunocaecal anastomosis; RDD, right dorsal displacement; LDD, left dorsal displacement; SC, small colon*

Compared with horses with an ileal impaction, the odds of late return to full feed (≥4 days) were greatest for horses with a small intestinal strangulating lesion that necessitated a resection (both jejunojejunal [OR 3.23; CI 2.06‐5.08; *P* < .001] and jejunocaecal [OR 4.97; CI 3.07‐8.04; *P* < .001] anastomoses), small colon lesion necessitating a resection and anastomosis (OR 4.45; CI 2.74‐7.23; *P* < .001) and caecal impaction (with by‐pass [OR 4.82; CI 2.93‐7.93; *P* < .001] and with typhlotomy only [OR 4.28; CI 2.65‐6.90; *P* < .001]). Whereas horses with a small intestinal strangulating lesion that did not necessitate a resection had an OR 1.85 (CI 1.20‐2.86; *P* = .006) and large colon torsion had an OR 1.78 (CI 1.13‐2.80; *P* = .01), when compared with horses with an ileal impaction. Horses with a large colon displacement had decreased odds of late return to full feed (OR 0.41; CI 0.25‐0.69; *P* = .001) (Figure 3).

### 3.4 Addition of supplements

Twenty‐six per cent of respondents reported using probiotics for ≥360° colon torsions; however, they were used less frequently following other intestinal lesions (13%‐22%). Prebiotics were not widely used in any post‐operative colic case (2%‐4%). Electrolytes were used by responders for 9%‐14% of all lesions and salt for 11%‐17%. Mineral oils were specifically mentioned for small colon resection and anastomosis (45% of respondents), and also for caecal impactions with typhlotomy (26%). Mineral oils were given sporadically post‐operatively following correction of other lesions (13%‐21%) (Data S2).

### 3.5 Approaches following cessation of post‐operative reflux (POR) to re‐introduction of water and feed

The answers to this open‐ended question were grouped into categories. Water re‐introduction was categorised as restricted (<2 L), reduced (ie < 10 L) and free choice. Results indicated that 56% (n = 77/137) of those who answered this question would restrict the water offered initially, 12% (n = 16/137) would offer a reduced amount (ie < 10 L) and 6% (n = 8/137) would offer free choice water. It was not possible to clearly identify the volume of water that would be offered initially by the remaining 26% (n = 36/137) of respondents. Feeding was initially started with small quantities with grass (42%; n = 57/137), complete, pelleted diet (27%; n = 37/137) and hay (18%; n = 24/137). There were difficulties in categorising the respondents’ answers regarding how early re‐feeding would be initiated following cessation of reflux, and this was not always clearly stated in the free‐text, open‐ended answers. For the majority of respondents, however, this was immediately (50%; n = 69/137) with a smaller number of respondents indicating a delay by 12‐24 hours (28%; n = 38/137) in initiating re‐feeding following cessation of reflux. A small proportion of respondents (4%; n = 6/137) indicated that they would use an ultrasound‐guided approach to the re‐introduction of feeding.

## 4 DISCUSSION

This post‐operative colic nutrition survey is the first to describe current clinical practice. The results highlight that there is variation in the approaches to re‐feeding post‐surgical colic cases and this is often lesion dependent. The findings of this study represent a description of current practice by veterinary specialists and does not provide evidence for feeding recommendations. The information obtained from a cross‐sectional study, such as this, is often the first step providing the impetus to generating higher levels of hierarchy evidence.^1^, ^2^ International surveys of clinical practice are an established approach used in human medicine when there is insufficient data for evidence‐based guidelines.^9^ Moreover, the data may be a starting point for a Delphi process, which is a tool widely used for developing a consensus in human medicine. This strategy may be particularly useful with the relative paucity of clinical evidence surrounding the topic of equine post‐operative colic nutrition.

Published reference texts have suggested that uncomplicated surgical cases of intestinal displacements without an enterotomy/anastomosis may begin re‐introduction of EN earlier than horses that have required a small intestinal resection and anastomosis.^8^ Among peer‐reviewed papers from single‐centre studies, there is a broad range of reported fasting times for cases that have required small intestinal resection and anastomosis, ranging from 18 to 24 hours in an American university referral hospital,^12^ a mean of 76 hours in a UK private referral hospital,^13^ up to fasting periods of > 10 days in some cases affected by post‐operative ileus (POI).^14^ Our survey results provide a greater understanding of current veterinary specialist practice indicating the time period over which horses are re‐introduced feed and water for a variety of common intestinal lesions in uncomplicated cases.

Most respondents reported that they would re‐introduce restricted water <2 L for small intestinal lesions. Re‐introduction of feed would be initiated later (≥24 hours following recovery from anaesthesia), and they tended to be returned to full rations of feed later (≥4 days once re‐feeding has begun) compared with many other intestinal lesions. Most respondents indicated that they would instigate re‐introduction of feed with handfuls of grass or a complete, pelleted feed.

Fasting following surgery, in theory, allows healing and protection, and reduces the risks of dehiscence, peritonitis, impactions and ileus; however, there is no evidence to support starvation post‐operatively, and no benefit has been demonstrated in people.^3^, ^4^, ^5^, ^6^, ^7^ In the initial post‐operative period, it has been anecdotally suggested in the referenced textbooks that restricting the amount of feed may minimalise potential deleterious effects on the anastomosis site.^8^ In uncomplicated equine cases, trophic feeding (trickle feeding small amounts initially) is performed and advocated, whereby grass and soft feeds (eg bran mashes or soaked fibre cubes) are first introduced followed by hay, with the quantity being gradually increased,^15^ as indicated and supported by the survey results.

For large intestinal lesions, respondents indicated that the re‐introduction of water was usually free choice and earlier (<12 hours following recovery from anaesthesia) compared with lesions elsewhere in the intestinal tract. Feeding was generally performed earlier (<24 hours following recovery from anaesthesia) and clinicians were more inclined to feed larger quantities following the correction of large intestinal lesions (especially uncomplicated cases of intestinal displacements, whereas large colon torsions were offered feed later) in comparison to small intestinal lesions. Once re‐introduction of feed had begun, the horses were returned to full feeds sooner as indicated in the survey. Hay has been considered important, especially following surgery for correction of large colon displacements,^8^ and the survey respondents also indicated this preference. The risk of diarrhoea following celiotomy for large intestinal lesions appeared to be greater^16^ and good‐quality forage has been considered essential in these cases.^8^

There are few reports in the literature regarding the re‐introduction of feed and water following resolution of caecal impactions, either with a typhlotomy or by‐pass procedure. Aitken et al. (2015) demonstrated in a single‐centre, retrospective case series that re‐feeding did not differ between surgically or medically treated caecal impactions with the median time to first feed being 36 hours and median time to first hay being 72 hours.^17^ There may be a variation of answers from respondents regarding re‐introduction of feed and water for caecal impactions due to the sparse information in the reference texts.^17^ Perhaps this is a less common lesion encountered by veterinary specialists. The frequency or relative frequency that the veterinary specialist encounters these surgical lesions was not ascertained in the survey.

For small colon strangulation, the starvation period was often longer compared with other lesions, and the time to reach full feeds was delayed. This delay indicated by the survey respondents maybe explained by the common perception that surgery for the correction of small colon disorders has more potential complications, such as increased risk of developing diarrhoea, when compared with surgical controls.^18^ Those horses necessitating a resection and anastomosis of the small colon had a reduced long‐term survival.^18^ Other hypothesised reasons for possible increased complications have been anecdotally stated in referenced text due to restricted surgical access, high bacterial content and the presence of coarse faeces.^8^ Therefore, low‐bulk, soft rations and mineral oil/laxatives are considered key in the initial management of these cases to minimise distension at the colotomy or anastomosis site.^8^

This survey indicates that early EN is frequently practised in large colon displacements, but is less commonly undertaken for other lesions such as small intestinal strangulating lesions. The positive effects of EN in recovery and survival for the post‐operative colic have for the majority been extrapolated from the human literature^3^, ^4^, ^5^, ^6^, ^7^ and discussed within the referenced textbooks.^8^, ^15^ In a small single‐centre study of 37 horses, Valle et al. (2019) identified an association with feeding and recovery time in equine colic cases after laparotomy.^19^ Those that were consuming forage within 12 hours post‐operatively had a shorter recovery time.^19^ Although the authors recognise the fact that horses that recover swiftly after surgery are likely to be offered EN faster than those that recover more slowly, they also suggest that EN can positively affect intestinal motility and enterocyte function to provide a beneficial effect.^19^

Supplements were used infrequently for the majority of intestinal lesions in the post‐operative period. However, a greater number of respondents indicated that they would use a probiotic for large colon torsions, and mineral oils were used commonly for small colon lesions. There are minimal studies and equivocal evidence for the clinical use of probiotics in equine gastrointestinal diseases (reviewed by Schoster et al.^20^). The clinical benefit has not been assessed in cases of large colon torsions; there are a small number of studies assessing their benefit in acute enterocolitis,^21^, ^22^ foal diarrhoea^23^, ^24^ and for salmonella shedding.^25^, ^26^, ^27^ There were limitations to the analysis of these data since not all respondents answered this question, and it was, therefore, unclear whether this item omission was because the respondents were not routinely using supplements. This was classed as missing/unanswered data. Therefore, it is possible that the true number of ‘no/none’ answers would be higher than reported here.

Intestinal dysmotility is one of the predominant concerns following equine gastrointestinal surgery, especially when small intestinal resection and anastomosis are performed. Cases of POR require intravenous fluid therapy and frequent nasogastric intubation to decompress the stomach; in such cases, parenteral nutrition may be indicated if reflux/starvation persists for >2‐3 days.^13^, ^28^, ^29^ In some circumstances, a repeat laparotomy may be necessary if POR persists.^30^ The survey respondents indicated that following cessation of POR, restricted volumes of water are offered initially, followed by feeding with small quantities of predominantly grass and/or a complete, pelleted diet. A proportion of respondents indicated that they employed an ultrasound‐guided approach to the re‐introduction of water and feed, by assessing the stomach size, duodenal contractility and evidence of distended small intestines. In our nutrition survey, as well as a recent survey by Lefebvre et al.^10^, ^11^ regarding POI, respondents expressed the utility of ultrasound evidence of distended small intestines, small intestinal motility and duodenal contractility. There is minimal published evidence behind the ultrasound‐guided approach to assessing POR^31^ or the use of ultrasonography to guide re‐feeding, but the use for monitoring treatment of colic has been described.^32^, ^33^

The survey had a low response rate, albeit similar to other surveys targeting equine specialists.^10^, ^11^, ^34^ This low response rate could lead to nonresponse bias; however, the effect of this bias is difficult to ascertain since the anonymous nature of the survey precluded the analysis of the nonresponders. However, the low response rate may indicate that a portion of specialists may not be practicing veterinarians or may not be managing post‐operative colic cases. Therefore, the portion of respondents who have answered the survey may have facilitated a more accurate representation of current clinical practice. Alternatively, the low response rate may reflect a portion of clinicians who do see these cases but did not have time to respond. Individual responses were requested; however, it was possible that some practices were represented by only one specialist within the hospital. This may have also contributed to the low response rates. Results of this survey describe current clinical practice, which does not necessarily reflect the optimal approach. There is currently no scientific evidence regarding nutritional management to increase survival and reduce complications following colic surgery. Perhaps the results from this survey will stimulate further research that could look to investigate nutritional strategies in post‐operative colic cases.

## 5 CONCLUSIONS

This study identified that there are differing approaches to re‐feeding post‐surgical colic cases dependent on the lesion. Broadly, there were heterogeneous answers from the specialist respondents regarding the re‐introduction of feed and water. However, clearer tendencies for earlier or later re‐introduced feed and water for different gastrointestinal lesions can be observed when evaluating the binary logistic regression results.

## CONFLICT OF INTERESTS

No competing interests have been declared.

## AUTHORS CONTRIBUTIONS

A. Lawson, C. Sherlock and T. Mair contributed to the study design, data analysis and interpretation, preparation of the manuscript and final approval of the manuscript. J. Ireland contributed to the data analysis and interpretation, preparation of the manuscript and final approval of the manuscript.

## ETHICAL ANIMAL RESEARCH

Bell Equine Veterinary Clinic's Ethical Review Committee approved this study.

## OWNER INFORMED CONSENT

Completion of the questionnaire was taken as participant consent.

## Supporting information

Supplementary Material

Supplementary Material

## Data Availability

The data that support the findings of this study are available from the corresponding author upon reasonable request.

## References

[1] Mann CJ. Observational research methods. Research design II: cohort, cross sectional, and case‐control studies. Emerg Med J. 2003;20(1):54–60. 12533370 10.1136/emj.20.1.54 PMC1726024

[2] Dalkey N, Helmer O. An experimental application of the Delphi method to the use of experts. Manag Sci. 1963;9(3):458–67.

[3] Lewis SJ, Egger M, Sylvester PA, Thomas S. Early enteral feeding versus ‘nil by mouth’ after gastrointestinal surgery: systematic review and meta‐analysis of controlled trials. Br Med J. 2001;323:773–6. 11588077 10.1136/bmj.323.7316.773 PMC57351

[4] Andersen HK, Lewis SJ, Thomas S. Early enteral nutrition within 24h of colorectal surgery versus later commencement of feeding for postoperative complications. Cochrane Database Syst Rev. 2006;4:CD004080. 10.1002/14651858.CD004080.pub2 17054196

[5] Lewis SJ, Andersen HK, Thomas S. Early enteral nutrition within 24 h of intestinal surgery versus later commencement of feeding: a systematic review and meta‐analysis. J Gastrointest Surg. 2008;13:569–75. 18629592 10.1007/s11605-008-0592-x

[6] Osland E, Yunus RM, Khan S, Memon MA. Early versus traditional postoperative feeding in patients undergoing resectional gastrointestinal surgery: a meta‐analysis. J Parenter Enter Nutr. 2011;35(4):473–87. 10.1177/0148607110385698 21628607

[7] Herbert G, Perry R, Andersen HK, Atkinson C, Penfold C, Lewis SJ, et al. Early enteral nutrition within 24 hours of lower gastrointestinal surgery versus later commencement for length of hospital stay and postoperative complications. Cochrane Database Syst Rev. 2019;7:CD004080. 10.1002/14651858.CD004080.pub4 31329285 PMC6645186

[8] Mair TS. Feeding management pre‐ and post‐surgery. In: Geor RJ, Harris PA, Coenen M. Equine Applied and Clinical Nutrition. Edinburgh: Saunders Elsevier, 2013;607–17.

[9] Martin D, Joliat GR, Halkic N, Demartines N, Schäfer M. Perioperative nutritional management of patients undergoing pancreatoduodenectomy: an international survey among surgeons. HPB. 2020;22(1):75–82. 31257012 10.1016/j.hpb.2019.05.009

[10] Lefebvre D, Pirie RS, Handel IG, Tremaine WH, Hudson NPH. Clinical features and management of equine post operative ileus: Survey of diplomates of the European Colleges of Equine Internal Medicine (ECEIM) and Veterinary Surgeons (ECVS). Equine Vet J. 2016;48(2):182–7. 25256601 10.1111/evj.12355

[11] Lefebvre D, Hudson NPH, Elce YA, Blikslager A, Divers TJ, Handel IG, et al. Clinical features and management of equine post operative ileus (POI): Survey of Diplomates of the American Colleges of Veterinary Internal Medicine (ACVIM), Veterinary Surgeons (ACVS) and Veterinary Emergency and Critical Care (ACVECC). Equine Vet J. 2016;48(6):714–9. 26502215 10.1111/evj.12520

[12] Freeman DE, Hammock P, Baker GJ, Goetz T, Foreman JH, Schaeffer DJ, et al. Short‐and long‐term survival and prevalence of postoperative ileus after small intestinal surgery in the horse. Equine Vet J. 2000;32:42–51. 10.1111/j.2042-3306.2000.tb05333.x 11202382

[13] Durham A, Phillips T, Walmsley J, Newton J. Study of the clinical effects of postoperative parenteral nutrition in 15 horses. Vet Rec. 2003;153:493–8. 14601796 10.1136/vr.153.16.493

[14] Cohen N, Lester G, Sanchez L, Merritt A, Roussel AJ. Evaluation of risk factors associated with development of postoperative ileus in horses. J Am Vet Med Assoc. 2004;225:1070–8. 15515986 10.2460/javma.2004.225.1070

[15] Pratt‐Phillips SE, Geor RJ. Nutritional management of the colic patient. In: Blikslager AT, White NA, Moore JN, Mair TS. The Equine Acute Abdomen. 3rd ed. Hoboken: Wiley‐Blackwell, 2017;491–508.

[16] Cohen ND, Honnas CM. Risk factors associated with development of diarrhea in horses after celiotomy for colic: 190 cases (1990–1994). J Am Vet Med Assoc. 1996;209(4):810–3. 8756885

[17] Aitken MR, Southwood LL, Ross BM, Ross MW. Outcome of surgical and medical management of cecal impaction in 150 horses (1991–2011). Vet Surg. 2015;44(5):540–6. 25302715 10.1111/j.1532-950X.2014.12286.x

[18] De Bont MP, Proudman CJ, Archer DC. Surgical lesions of the small colon and post operative survival in a UK hospital population. Equine Vet J. 2013;45(4):460–4. 23173766 10.1111/evj.12005

[19] Valle E, Giusto G, Penazzi L, Giribaldi M, Bergero D, Fradinho MJ, et al. Preliminary results on the association with feeding and recovery length in equine colic patients after laparotomy. J Anim Physiol Anim Nutr. 2019;103(4):1233–41. 10.1111/jpn.13102 31025443

[20] Schoster A, Weese JS, Guardabassi L. Probiotic Use in Horses – What is the Evidence for Their Clinical Efficacy? J Vet Intern Med. 2014;28:1640–52. 25231539 10.1111/jvim.12451 PMC4895607

[21] Desrochers AM, Dolente BA, Roy MF, Boston R, Carlisle S. Efficacy of Saccharomyces boulardii for treatment of horses with acute enterocolitis. J Am Vet Med Assoc. 2005;227:954–9. 16190596 10.2460/javma.2005.227.954

[22] Boyle AG, Magdesian KG, Gallop R, Sigdel S, Durando MM. Saccharomyces boulardii viability and efficacy in horses with antimicrobial‐induced diarrhoea. Vet Rec. 2013;172(5):128. 23161811 10.1136/vr.100833

[23] Weese JS, Rousseau J. Evaluation of Lactobacillus pentosus WE7 for prevention of diarrhea in neonatal foals. J Am Vet Med Assoc. 2005;226:2031–4. 15989186 10.2460/javma.2005.226.2031

[24] Ströbel C, Günther E, Romanowski K, Büsing K, Urubschurov V, Zeyner A. Effects of oral supplementation of probiotic strains of Lactobacillus rhamnosus and Enterococcus faecium on diarrhoea events of foals in their first weeks of life. J Anim Physiol Anim Nutr. 2018;102:1357–65. 10.1111/jpn.12923 29790614

[25] Parraga ME, Spier SJ, Thurmond M, Hirsh D. A clinical trial of probiotic administration for prevention of Salmonella shedding in the postoperative period in horses with colic. J Vet Intern Med. 1997;11:36–41. 9132482 10.1111/j.1939-1676.1997.tb00071.x

[26] Kim LM, Morley PS, Traub‐Dargatz JL, Salman MD, Gentry‐Weeks C. Factors associated with Salmonella shedding among equine colic patients at a veterinary teaching hospital. J Am Vet Med Assoc. 2001;218:740–8. 11280409 10.2460/javma.2001.218.740

[27] Ward MP, Alinovi CA, Couetil LL, Glickman LT, Wu CC. A randomized clinical trial using probiotics to prevent Salmonella fecal shedding in hospitalized horses. J Equine Vet Sci. 2004;24:242–7.

[28] Lopes M, White N. Parenteral nutrition for horses with gastrointestinal disease: a retrospective study of 79 cases. Equine Vet J. 2002;34:250–7. 12108742 10.2746/042516402776186083

[29] Durham AE, Phillips TJ, Walmsley JP, Newton JR. Nutritional and clinicopathological effects of post‐operative parenteral nutrition following small intestinal resection and anastomosis in the mature horse. Equine Vet J. 2004;36:390–6. 15253078 10.2746/0425164044868369

[30] Bauck AG, Easley JT, Cleary OB, Graham S, Morton AJ, Rötting AK, et al. Response to early repeat celiotomy in horses after a surgical treatment of jejunal strangulation. Vet Surg. 2017;46(6):843–50. 28556997 10.1111/vsu.12670

[31] Lawson AL, Sherlock CE, Mair TS. Equine duodenal motility, assessed by ultrasonography, as a predictor of reflux and survival following colic surgery. Equine Vet Educ. 2021;33:84–9.

[32] Desrochers A. Imaging of the abdomen. In: Blikslager AT, White NA, Moore JN, Mair TS. The Equine Acute Abdomen. 3rd ed. Hoboken: Wiley‐Blackwell, 2017;271–84.

[33] Mair TS. Monitoring treatment for abdominal disease. In: Blikslager AT, White NA, Moore JN, Mair TS. The Equine Acute Abdomen. 3rd ed. Hoboken: Wiley‐Blackwell, 2017;613–23.

[34] Christley RM. Questionnaire survey response rates in equine research. Equine Vet J. 2016;48(2):138–9. 26820584 10.1111/evj.12552

