# Supplementary material for: Equine nutrition in the post‐operative colic: Survey of Diplomates of the American Colleges of Veterinary Internal Medicine and Veterinary Surgeons, and European Colleges of Equine Internal Medicine and Veterinary Surgeons
Source: Equine Vet J. 2021 Jan 9;53(5):1015–24. doi: 10.1111/evj.13381 (PMC8451781; doi:10.1111/evj.13381)
Supplement: Supplementary file 1 — Supplementary Material [file EVJ-53-1015-s001.pdf]

Supplementary Item 1: Preview view of the survey questionnaire designed within Survey Monkey, a web-based proprietary software. The survey consisted of 10 common surgical intestinal lesions for each scenario, 7 identical questions were asked; both closed (questions 1-6) and open-ended questions (question 7). A final question was posed regarding the approaches to re-introduction of feed and water following cessation of post-operative reflux.

Post-operative colic nutrition

Personal Details

We are interested in your approach/ protocol for re-introducing feed and water to horses following colic surgery when considering different surgical lesions. The information will be used for research purposes and your answers will remain anonymous. If you would like a summary of the final pooled results, please make sure you enter your name and email address.

OK

1. Full Name

* 2. Credentials

- ☐ DipACVS
- ☐ DipACVIM
- ☐ DipECVS
- ☐ DipECEIM

Other (please specify)

3. Email address

NEXT

Ileal impaction - no enterotomy, no resection/by-pass

1. When would you offer water following anaesthesia (assuming no reflux)?

If ≥ 24 hours, please state.

2. Volume of water offered first?

Other (please specify)

3. When would you first offer feed following recovery from anaesthesia?

If ≥ 48 hours, or if you make decision on feeding based on faecal output or other factors, please specify.

4. What type of feed would you offer when re-introducing feeding?

Please describe your routine feeding protocol.

5. Please estimate quantity of feed you would offer initially.

Other (please specify)

6. Once re-feeding has begun, over how many days do you aim for the horse to return to full feed (assuming no reflux/ complications)?

If > 7 days, please state.

7. Addition of any other supplements? e.g. electrolytes, prebiotics, probiotics, salt, mineral oil, etc.

PREV NEXT

Small intestinal strangulation without resection

1. When would you offer water following anaesthesia (assuming no reflux)?

If ≥ 24 hours, please state.

2. Volume of water offered first?

Other (please specify)

3. When would you first offer feed following recovery from anaesthesia?

If ≥ 48 hours, or if you make decision on feeding based on faecal output or other factors, please specify.

4. What type of feed would you offer when re-introducing feeding?

Please describe your routine feeding protocol.

5. Please estimate quantity of feed you would offer initially.

Other (please specify)

6. Once re-feeding has begun, over how many days do you aim for the horse to return to full feed (assuming no reflux/ complications)?

If > 7 days, please state.

7. Addition of any other supplements? e.g. electrolytes, prebiotics, probiotics, salt, mineral oil, etc.

PREV NEXT

Small intestinal strangulation with 1m resection - Jejunojejunal anastomosis

1. When would you offer water following anaesthesia (assuming no reflux)?

If ≥ 24 hours, please state.

2. Volume of water offered first?

Other (please specify)

3. When would you first offer feed following recovery from anaesthesia?

If ≥ 48 hours, or if you make decision on feeding based on faecal output or other factors, please specify.

4. What type of feed would you offer when re-introducing feeding?

Please describe your routine feeding protocol.

5. Please estimate quantity of feed you would offer initially.

Other (please specify)

6. Once re-feeding has begun, over how many days do you aim for the horse to return to full feed (assuming no reflux/ complications)?

If > 7 days, please state.

7. Addition of any other supplements? e.g. electrolytes, prebiotics, probiotics, salt, mineral oil, etc.

PREV NEXT

Small intestinal strangulation with 1m resection - Jejunocaecal anastomosis

1. When would you offer water following anaesthesia (assuming no reflux)?

If ≥ 24 hours, please state.

2. Volume of water offered first?

Other (please specify)

3. When would you first offer feed following recovery from anaesthesia?

If ≥ 48 hours, or if you make decision on feeding based on faecal output or other factors, please specify.

4. What type of feed would you offer when re-introducing feeding?

Please describe your routine feeding protocol.

5. Please estimate quantity of feed you would offer initially.

Other (please specify)

6. Once re-feeding has begun, over how many days do you aim for the horse to return to full feed (assuming no reflux/ complications)?

If > 7 days, please state.

7. Addition of any other supplements? e.g. electrolytes, prebiotics, probiotics, salt, mineral oil, etc.

PREV NEXT

Large colon displacement - left dorsal displacement / nephrosplenic entrapment

1. When would you offer water following anaesthesia (assuming no reflux)?

If ≥ 24 hours, please state.

2. Volume of water offered first?

Other (please specify)

3. When would you first offer feed following recovery from anaesthesia?

If ≥ 48 hours, or if you make decision on feeding based on faecal output or other factors, please specify.

4. What type of feed would you offer when re-introducing feeding?

Please describe your routine feeding protocol.

5. Please estimate quantity of feed you would offer initially.

Other (please specify)

6. Once re-feeding has begun, over how many days do you aim for the horse to return to full feed (assuming no reflux/ complications)?

If > 7 days, please state.

7. Addition of any other supplements? e.g. electrolytes, prebiotics, probiotics, salt, mineral oil, etc.

PREV NEXT

Large colon displacement - right dorsal displacement (no resection)

1. When would you offer water following anaesthesia (assuming no reflux)?

If ≥ 24 hours, please state.

2. Volume of water offered first?

Other (please specify)

3. When would you first offer feed following recovery from anaesthesia?

If ≥ 48 hours, or if you make decision on feeding based on faecal output or other factors, please specify.

4. What type of feed would you offer when re-introducing feeding?

Please describe your routine feeding protocol.

5. Please estimate quantity of feed you would offer initially.

Other (please specify)

6. Once re-feeding has begun, over how many days do you aim for the horse to return to full feed (assuming no reflux/ complications)?

If > 7 days, please state.

7. Addition of any other supplements? e.g. electrolytes, prebiotics, probiotics, salt, mineral oil, etc.

PREV NEXT

≥360 degree large colon torsion - no resection

1. When would you offer water following anaesthesia (assuming no reflux)?

If ≥ 24 hours, please state.

2. Volume of water offered first?

Other (please specify)

3. When would you first offer feed following recovery from anaesthesia?

If ≥ 48 hours, or if you make decision on feeding based on faecal output or other factors, please specify.

4. What type of feed would you offer when re-introducing feeding?

Please describe your routine feeding protocol.

5. Please estimate quantity of feed you would offer initially.

Other (please specify)

6. Once re-feeding has begun, over how many days do you aim for the horse to return to full feed (assuming no reflux/ complications)?

If > 7 days, please state.

7. Addition of any other supplements? e.g. electrolytes, prebiotics, probiotics, salt, mineral oil, etc.

PREV NEXT

Caecal impaction - typhlotomy only

1. When would you offer water following anaesthesia (assuming no reflux)?

If ≥ 24 hours, please state.

2. Volume of water offered first?

Other (please specify)

3. When would you first offer feed following recovery from anaesthesia?

If ≥ 48 hours, or if you make decision on feeding based on faecal output or other factors, please specify.

4. What type of feed would you offer when re-introducing feeding?

Please describe your routine feeding protocol.

5. Please estimate quantity of feed you would offer initially.

Other (please specify)

6. Once re-feeding has begun, over how many days do you aim for the horse to return to full feed (assuming no reflux/ complications)?

If > 7 days, please state.

7. Addition of any other supplements? e.g. electrolytes, prebiotics, probiotics, salt, mineral oil, etc.

PREV NEXT

Caecal impaction with by-pass

1. When would you offer water following anaesthesia (assuming no reflux)?

If ≥ 24 hours, please state.

2. Volume of water offered first?

Other (please specify)

3. When would you first offer feed following recovery from anaesthesia?

If ≥ 48 hours, or if you make decision on feeding based on faecal output or other factors, please specify.

4. What type of feed would you offer when re-introducing feeding?

Please describe your routine feeding protocol.

5. Please estimate quantity of feed you would offer initially.

Other (please specify)

6. Once re-feeding has begun, over how many days do you aim for the horse to return to full feed (assuming no reflux/ complications)?

If > 7 days, please state.

7. Addition of any other supplements? e.g. electrolytes, prebiotics, probiotics, salt, mineral oil, etc.

PREV NEXT

Small colon strangulation - resection and end to end anastomosis

1. When would you offer water following anaesthesia (assuming no reflux)?

If ≥ 24 hours, please state.

2. Volume of water offered first?

Other (please specify)

3. When would you first offer feed following recovery from anaesthesia?

If ≥ 48 hours, or if you make decision on feeding based on faecal output or other factors, please specify.

4. What type of feed would you offer when re-introducing feeding?

Please describe your routine feeding protocol.

5. Please estimate quantity of feed you would offer initially.

Other (please specify)

6. Once re-feeding has begun, over how many days do you aim for the horse to return to full feed (assuming no reflux/ complications)?

If > 7 days, please state.

7. Addition of any other supplements? e.g. electrolytes, prebiotics, probiotics, salt, mineral oil, etc.

PREV NEXT

Following cessation of reflux

1. Following cessation of post-operative reflux, how would you approach re-introduction of water and feed?

PREV NEXT

Thank you

Thank you for completing our survey.

OK

PREV DONE
