# Supplementary material for: Equine nutrition in the post‐operative colic: Survey of Diplomates of the American Colleges of Veterinary Internal Medicine and Veterinary Surgeons, and European Colleges of Equine Internal Medicine and Veterinary Surgeons
Source: Equine Vet J. 2021 Jan 9;53(5):1015–24. doi: 10.1111/evj.13381 (PMC8451781; doi:10.1111/evj.13381)
Supplement: Supplementary file 2 — Supplementary Material [file EVJ-53-1015-s002.pdf]

Equine Veterinary Journal BEVA

Supplementary Item 2: The answers to the open ended question 'Addition of any other supplements? e.g. electrolytes, prebiotics, probiotics, salt, mineral oil, etc.' were grouped into prebiotics, probiotics, mineral oils, electrolytes, salt block and no/none. Each respondent may have chosen greater than one option. The percentages of respondents that mentioned the aforementioned supplements or answered no/none were totalled and the percentage calculated as a percentage of the total respondents. The results are displayed rounded to the closest whole number.

	Intestinal scenarios – number of respondents (% respondents)																			
	Ileal impaction		Small intestinal strangulation - no resection		Small intestinal strangulation resection J-J		Small intestinal strangulation resection J-C		Left dorsal displacement		Right dorsal displacement		≥360 degree large colon torsion		Caecal impaction - typhlotomy only		Caecal impaction with by-pass		Small colon strangulation and resection	
Prebiotics	4	*3%*	3	*2%*	3	*3%*	3	*3%*	2	*2%*	4	*4%*	4	*4%*	4	*4%*	2	*2%*	2	*2%*
Probiotics	26	*18%*	25	*20%*	22	*20%*	22	*21%*	16	*17%*	15	*16%*	25	*26%*	21	*22%*	18	*21%*	13	*13%*
Mineral oil	23	*16%*	16	*13%*	15	*14%*	16	*15%*	15	*16%*	14	*15%*	13	*14%*	24	*26%*	18	*21%*	44	*45%*
Electrolytes	13	*9%*	13	*11%*	13	*12%*	13	*12%*	11	*11%*	11	*12%*	10	*10%*	13	*14%*	9	*11%*	13	*13%*
Salt	24	*17%*	18	*15%*	14	*13%*	13	*12%*	12	*13%*	12	*13%*	11	*11%*	14	*15%*	12	*14%*	12	*12%*
No/None	68	*48%*	62	*50%*	53	*48%*	49	*47%*	54	*56%*	48	*51%*	44	*46%*	35	*37%*	38	*45%*	29	*30%*
TOTAL	142		123		110		105		96		95		96		94		85		98	

J-J, jejunojejunal anastomosis; J-C, jejunocaecal anastomosis.
